# Rehabilitation interventions to support return to work for women with breast cancer: a systematic review and meta-analysis

**DOI:** 10.1186/s12885-021-08613-x

**Published:** 2021-08-05

**Authors:** Naomi Algeo, Kathleen Bennett, Deirdre Connolly

**Affiliations:** 1 Discipline of Occupational Therapy, Trinity College Dublin, Dublin, Ireland; 2 Data Science Centre, Royal College of Surgeons in Ireland, Dublin, Ireland

**Keywords:** Employment, activities of daily living, survivorship, Quality of life, breast neoplasms

## Abstract

**Background:**

Research recommends the development and evaluation of interventions to support women with breast cancer in returning to, or managing, work. Despite this, there has historically been a paucity of rehabilitation interventions to support women with breast cancer to maintain or return to their work role. The aim of this systematic review was to examine key characteristics of rehabilitation interventions, and their effectiveness on work outcomes for women with breast cancer, compared to usual care.

**Methods:**

A systematic review was conducted of controlled studies of rehabilitation interventions with work outcomes for women with breast cancer. Six databases were systematically searched: EMBASE, Web of Science, MEDLINE (OVID), CINAHL, PsycINFO, and the Cochrane Central Register of Controlled Trials (CENTRAL). Results are presented either as pooled odds ratio (OR) or pooled effect size (hedges g) between groups, with 95% confidence intervals (CI). Narrative synthesis was conducted on intervention outcomes not suitable for meta-analysis.

**Results:**

Five thousand, five hundred and thirty-five studies were identified. Nine out of 28 abstracts met inclusion criteria. Heterogeneity of interventions and outcomes precluded meta-analysis for most outcomes. Of the interventions included in meta-analysis, no significant differences compared to usual care were found for sick leave (2 studies (12 months); OR 1.11 (95% CI: 0.66 to 1.87), number of sick days taken (2 studies (six months); difference in effect: − 0.08, (95% CI: − 0.48 to 0.38) or working hours (2 studies (12 months); 0.19, (95% CI: − 0.20 to 0.64). Only one study, with a multidisciplinary intervention, showed a significant difference for work outcomes when compared to usual care. Work-specific content featured in three interventions only, none of which provided conclusive evidence for improvement in work outcomes. Enhanced physical and psychological sequalae, and quality of life was observed in some studies.

**Conclusion:**

There remains a lack of effective and methodologically rigorous rehabilitation intervention studies for breast cancer survivors. The development and evaluation of effective rehabilitation interventions to support return to work is warranted.

**Supplementary Information:**

The online version contains supplementary material available at 10.1186/s12885-021-08613-x.

## Introduction

Breast cancer accounted for over two million new cases in 2018 worldwide [1]. Survival is increasing, largely due to advancing treatments and earlier detection, and is as high as 85–90% at five-years in developed countries [2–4]. In line with increasing survivorship, there is a focus on optimising quality of life (QoL), for those living with and beyond cancer, including return to work (RTW). RTW rates vary across cancer types, and are influenced by personal, societal, workplace, healthcare, and legislative systems [5]. Typically, the one-year time point can be a milestone, where the mean delay in RTW has previously been reported at 11.4 months, however varying rates have been reported [6, 7]. This could be related to disease and treatment-related factors which are often cited as RTW barriers, in addition to health-related QoL (HRQoL), cancer-related fatigue, cognitive dysfunction, and depression and anxiety [8–10]. Other disease and treatment-related factors often observed in those with breast cancer can impact on functional ability, including axillary web syndrome, changes in spinal alignment post-surgery, and lymphoedema [11–13]. Despite this, many disease and treatment-related factors are amenable to change through rehabilitation [14].

A Cochrane review, providing evidence for vocational interventions to support RTW, reported moderate-quality evidence for multidisciplinary interventions to enhance work outcomes for all cancer cohorts including breast cancer, yet found it ‘remarkable’ that there remains a paucity in vocational interventions [15]. Vocational interventions have previously demonstrated promising outcomes for those living with chronic conditions such as heart disease, mental health disorders and intellectual disabilities [16–18]. Despite potential to enhance work outcomes for women with breast cancer, a previous systematic review yielded only four intervention studies, three of which were uncontrolled [19]. The aim of this study, therefore, was to systematically review rehabilitation intervention studies for women with breast cancer in relation to content, delivery and effectiveness of interventions on at least one work outcome when compared to usual care. Outcome measurements and theoretical frameworks underpinning interventions were also explored.

## Methods

This review is reported as per Preferred Reporting Items for Systematic reviews and Meta-Analyses (PRISMA) checklist [20]. An initial review protocol was registered on the International Prospective Register of Systematic Reviews (PROSPERO) [ID: CRD42019145557] prior to commencing the review. However, as only one work-specific intervention study [21] was identified in the initial search, inclusion criteria were expanded to include rehabilitation interventions that measured the impact of the intervention on one or more work-related outcomes.

### Eligibility criteria

The following eligibility criteria were set:

#### Study designs

Experimental designs including randomised control trials (RCTs) and quasi-experimental designs (with a comparator) were included.

#### Participants

The population was limited to women who had a breast cancer diagnosis and were ≥ 18 years old.

#### Interventions

Any type of non-pharmacological intervention which aimed to rehabilitate women with breast cancer was included. Interventions could be group, individual and/or digital in format, and could be vocational, psychosocial, physical or multi-disciplinary (combination of vocational, psychosocial and/or physical) in nature.

#### Comparators

There were no limits on comparator.

#### Outcomes

Studies were included only if they reported a minimum of one work-related outcome (primary outcome). For example, working hours, RTW status, sick days, etc. Secondary outcomes included physical, psychological and quality of life outcomes.

### Information sources and search strategy

A search strategy was developed with a medical librarian, and applied to EMBASE, Web of Science, MEDLINE (OVID), CINAHL, PsycINFO, and the Cochrane Central Register of Controlled Trials. For further details, see Supplementary Material 1. Backwards and forwards chaining of all full-texts was also completed to identify reviews which were relevant but did not meet criteria for full-text review.

### Search selection

Abstracts and titles of retrieved studies were screened by one reviewer. Where uncertainty remained, the study was examined in the full-text review to determine eligibility. Three reviewers were involved in full-text review. Where disagreement occurred between two reviewers regarding article inclusion/exclusion, a third reviewer intervened. Once full-text review was complete, data were extracted from included studies. EndNote was used to manage all retrieved studies, and Covidence for screening and data extraction.

### Data collection process and data items extracted

A data extraction tool based on the Cochrane Handbook for Systematic Review of Interventions [22] was applied to independently extract data from each study onto an excel spreadsheet. Items recorded included:

- Author(s), year of publication
- Study-design, setting, inclusion/exclusion criteria
- Type of intervention: format, duration, content, and facilitators
- Comparator
- Theoretical framework (as per Medical Research Council framework for complex interventions [23]).
- Outcomes: primary (work) and secondary (physical, psychological, and QoL) outcomes, outcome measures, and follow-up periods.

### Risk of bias in individual studies

Two reviewers assessed risk of bias of each study using the Cochrane Handbook for Systematic Reviews of Interventions [22]. In cases of disagreement, the two reviewers discussed, with a third reviewer available for any unresolved disagreements.

### Summary measures

Where outcomes were continuous, the estimated effect size was calculated from each published study using mean differences and standard deviations from each group (intervention and control) to calculate a standardised effect size using Hedges *g* formula. For binary outcomes, odds ratios were used.

### Synthesis of results

A meta-analysis of primary and secondary outcomes was planned if sufficient information was available and the studies were not too heterogeneous in relation to interventions, study designs, outcomes and measures of effect. If statistical synthesis was not possible, a narrative synthesis was planned to be conducted.

### Heterogeneity and pooling (meta-analysis) across studies

I^2^ index was used for the percentage of variance in meta-analysis attributable to study heterogeneity. However, this should be interpreted cautiously when a meta-analysis has few studies and can provide substantial bias, in which case confidence intervals (CIs) should supplement biased point estimate I^2^ [24]. The H^2^ statistic was also examined, where 1 is equal to perfect study homogeneity. The H^2^ statistic was considered where there were common measures across studies that could be pooled. In the case of binary outcomes, odds ratios (OR) and 95% CIs were extracted or calculated for each study from available data. In the presence of significant heterogeneity, meta-analysis was performed using a random effects approach. Penalised likelihood is used for computing 95% confidence intervals for continuous measures. For pooling ORs the peto method was used for fixed (or random) effects.

## Results

**Fig. 1:**
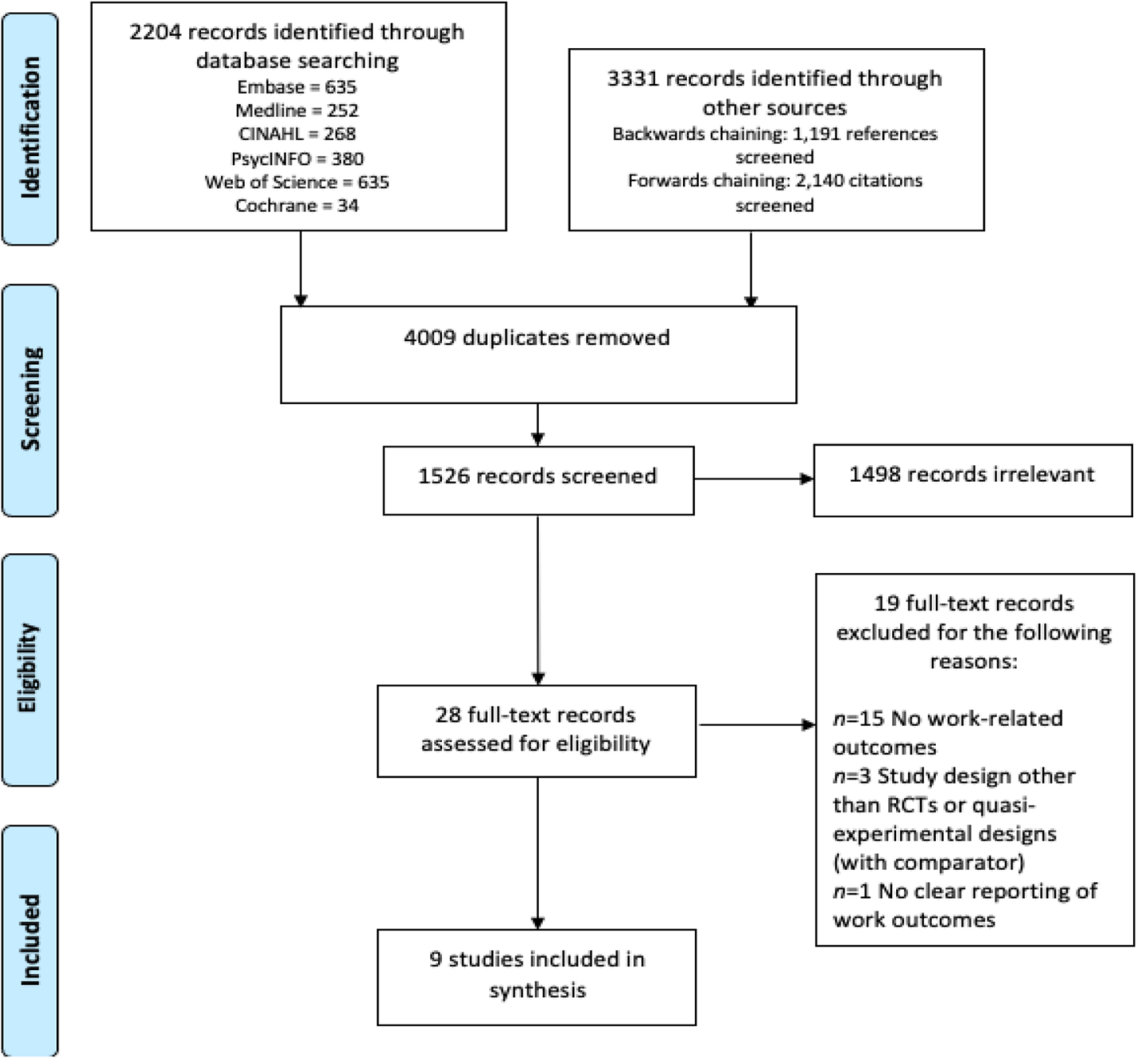
PRISMA Flow Diagram

Using the search strategy, 5535 records were identified, of which 28 papers met the inclusion criteria for full-text review (Fig. 1). Nine of the 28 studies were included in final synthesis. Of the 19 excluded papers, reasons for exclusion included (i) no work outcomes (*n* = 15), (ii) study-design other than RCTs or quasi-experimental designs (with comparator) (*n* = 3), and (iii) no clear reporting of work outcomes (*n* = 1). Further detail on studies excluded can be found in Supplementary Material 2.

### Study characteristics

Of the nine included studies, all were RCT in design, three of which were pilot RCTs (Table 1) [21, 25, 26]. Most studies (*n* = 6) were published since 2010 with the remaining three studies spread across the 1980s [27], 1990s [28], and 2000s [26]. Six studies were set in Europe [21, 27, 29–32], two in Canada [25, 28], and one in the United States [26]. Most interventions were delivered in a hospital setting (*n* = 6). One study did not specify context of intervention delivery however indicated that the intervention was partially home-based [26].

**Table 1:** Study and Participant Characteristics of included studies

Study Characteristics					Participant Characteristics		
Author	Year	Design	Country	Setting	***N*** (at allocation)	Inclusion Criteria	Age
**Björneklett et al.**	2013	RCT	Sweden	Resort (type not specified)	382 Intervention =191 Control =191	- Newly diagnosed primary breast cancer - No previous malignancy - The physical and mental capability to participate in group interventions and to fill in questionnaire - Expected survival time of > 12 months - Analyses limited to those under the age of 65 years old.	Overall = Unknown Intervention = 57.8 Control = 58.7
**Bolam et al.**	2019	RCT	Sweden	Hospital	240 RT-HIIT^1^ = 79 AT-HIIT^2^ = 80 Control = 81	- Women - 18–70 years - Stage I-IIIa breast cancer - Scheduled to receive chemotherapy directly	Overall: Unknown RT-HIIT = 52.7 AT-HIIT = 54.4 Control = 52.6
**Hubbard et al.**	2013	Pilot RCT	Scotland, UK	Hospital and Community	22 Intervention = 8 Control =14	- 18–65 years - In paid employment or self-employed - Living or working in Lothian or Tayside, Scotland, UK - Diagnosed with invasive breast cancer tumour or ductal carcinoma in situ - Treated first with surgery	Overall = 50.5 Intervention = 49.7 Control = 51.0
**Ibrahim et al.**	2017	Pilot RCT	Canada	Community (Cancer Support Centre)	59 Intervention = 29 Control = 30	- Stage I-III breast cancer - 18–45 years - Scheduled to receive post-operative adjuvant treatment - Have an ECOG performance status 0–1.	Overall = 39.2 Intervention and Control = Unknown
**Jong et al.**	2018	RCT	The Netherlands	Hospital and Home	83 Intervention = 47 Control = 36	- Women between 18 and 70 years - Stage I–III breast cancer - Scheduled for (neo) adjuvant chemotherapy - Able to understand and speak Dutch - Phone and internet access	Overall: Unknown Intervention = 51 Control = 51
^1^RT-HIIT = Resistance Exercise and High-Intensity Interval Training; ^2^AT-HIIT = Moderate Intensity Aerobic Exercise and High-Intensity Interval Training							
**Study Characteristics**					**Participant Characteristics**		
**Author**	**Year**	**Design**	**Country**	**Setting**	***N*** **(at allocation)**	**Inclusion Criteria**	**Age**
**Maguire et al.**	1983	RCT	England, UK	Hospital (Inpatient Surgical Unit)	172 Intervention = Unknown Control = Unknown	- Women admitted for modified radical mastectomy with full axillary clearance.	Unknown
**Maunsell et al.**	1996	RCT	Canada	Hospital	250 Intervention = 123 Control =127	- Newly diagnosed breast cancer patients with localised or regional stage disease.	Overall: Unknown Intervention = 54.6; Control = 56.3
**Mourgues et al.**	2014	RCT	France	Hospital	232 Intervention = 117 Control =115	- Complete remission of invasive non-metastatic breast carcinoma - < 9 months after completion of chemotherapy/radiotherapy - No contraindication for physical activities - No cognitive disorders - Body mass index between 18.5–40 kg/m^2^	Overall: Unknown Intervention = 51.9 Control =51.9
**Rogers et al.**	2009	Pilot RCT	USA	Unknown and Home	41 Intervention = 21 Control =20	- English-speaking female - 18–70 years - Diagnosis of stage I, II, or IIIA. - Currently taking aromatase inhibitors or selective oestrogen receptor modulators and expected to remain on hormonal therapy for study duration (> 8 months) - Medical clearance. - If surgical procedure undertaken, enrolment delayed > 8 weeks post procedure.	Overall: 53; Intervention = 52 Control =54

### Participants

While all studies included women with breast cancer as an inclusion criterion, there were variations in eligibility criteria, including age (Table 1). Of the six studies specifying age as an inclusion criterion, three studies included 18–70-year-olds [26, 30, 31]. Four studies specified a staging criteria of stages I-III [25, 26, 30, 31]. Overall sample sizes ranged from 22 to 382 at allocation, [21, 29]. Participant baseline characteristics varied in value. Mean age in the intervention and control groups across the seven studies which specified it, ranged from 49.7–57.8 years and 51.0–58.7 years, respectively.

### Intervention characteristics

#### Content

Intervention content varied widely (Table 2). The majority of studies delivered a combination of physical and psychosocial interventions (*n* = 6) [21, 26, 27, 29, 31, 32]. Two studies delivered physical interventions [25, 30] and one study a psychosocial intervention [28]. Only three interventions delivered work-focused components to the intervention including specific vocational guidance, encouragement to RTW and information on sick leave and insurance [21, 27, 29].

**Table 2:** Intervention Characteristics and Outcomes of included studies

Author	Intervention Characteristics					
	Format	Content	Facilitator(s)	Duration	Theoretical Framework	Outcome measures
**Björneklett et al. (2013)**	Face-to-face Group.	**Physical/Psychosocial:** An information-based programme supplemented with relaxation, qigong, liberating dance, and social activities. Information sessions included: - Psychological reactions to serious disease, & coping strategies. - Practicalities of sick leave from work, insurance & impact of illness on finance - Food and nutrition	Oncologists, social workers, a psychologist, an art therapist, massage therapists, a dietician and a person trained in qigong and mental visualisation.	One-week inpatient stay followed by four-day follow-up two months later. Duration of individual sessions not specified.	None	***Sick Leave:*** Single item question (Yes/No) and number of days taken for sick leave. ***Health care utilisation:*** Asked the frequency and types of healthcare visits. ***Cost-effectiveness*** *Measured at:* - 2 months - 6 months - 12 months
**Bolam et al. (2019)**	Face-to-face Group.	**Physical:** **RT-HIIT**^1^: Resistance Exercises using machine and free weights followed by High Intensity Interval Training on a cycle ergometer. **AT-HIIT**^2^**:** 20 min of moderate intensity continuous Aerobic Exercise followed by HIIT on a cycle ergometer.	Exercise physiologist, oncology nurse.	60-min sessions twice per week on non-consecutive weekdays, over 16 weeks.	None	***Sick leave:*** Single item question (% of leave taken; 0, 25, 50, 75, 100%) ***Cancer-related fatigue:*** Revised Piper Fatigue Scale (PFS) ***Quality of Life:*** EORTC-QLQ-C30^1^ ***Symptom and Symptom Burden:*** Memorial Symptom Assessment Scale (MSAS) *Measured at:* - 1 Year - 2 Years
**Hubbard et al. (2013)**	Individual Face-to-face, Telephone	**Physical/Psychosocial:** Tailored Vocational Rehabilitation Case management. Based on assessment, participants were signposted to at least one of the following services: occupational therapy, physiotherapy, counsellor, psychology, occupational health nurse, and/or complementary therapy.	Case manager, occupational therapist, physiotherapist, counsellor, psychology, occupational health nurse, and complementary therapist	No set duration as interventions varied.	Bio-psychosocial model	***Sick leave:*** Self-report questionnaire (days) ***Employment:*** Questionnaire inc. left or remained in work, job role, hours worked ***Quality of Life:*** Functional Assessment of Cancer Therapy-Breast Cancer (FACT-B) [Version 4] and Breast Cancer Subscale. ***Cancer-related fatigue:*** Functional Assessment of Chronic Illness Therapy- Fatigue Scale (FACIT-F) *Measured at:* - 6 months - 12 months
^1^RT-HIIT = Resistance Exercise and High-Intensity Interval Training; ^2^AT-HIIT = Moderate Intensity Aerobic Exercise and High-Intensity Interval Training						
**Author**	**Intervention Characteristics**					**Outcome measures**
	**Format**	**Content**	**Facilitator(s)**	**Duration**	**Theoretical Framework**	
**Ibrahim et al. (2017)**	Individual Face-to-face (and encouragement for home exercises)	**Physical:** One-to-one teaching session supervised by exercise physiologist. Cardiovascular exercise, strength training, endurance programme, stretching programme	Exercise physiologist	Encouraged to perform the programme 2–3 times/week over 12 weeks.	None	***Working hours****:* Post hoc questionnaire ***Upper limb function:*** The Disability of Arm, Shoulder and Hand (DASH) *Measured at:* - Baseline (pre-radiation), - post-radiation - 3, 6, 12, and 18-months post-radiation
**Jong et al. (2018)**	Face-to-Face and Home-Based work | Group	**Physical/Psychosocial** A Dru-based Yoga. Programme which includes 15-min blocks of the following: - Breathing awareness - Energy block release - Body awareness - Relaxation In addition, women were provided a CD/MP3 download with 20-min relaxation and breathing exercises to complete at home.	Yoga instructors	75-min sessions **o**nce a week for 12 weeks.	None	***Reintegration to work:*** Assessed via telephone interview. Returned to work: Binary Yes/No. ***Fatigue:*** Multidimensional Fatigue Inventory [MFI]; Fatigue Quality List [FQL] ***Quality of Life:*** EORTC-QLQ-C-30^1^ ***Psychological Distress:*** Hospital Anxiety Depression Scale [HADS]; Impact of Events Scale [IES] ***Treatment expectations:*** Participants Expectations questionnaire. *Measured at:* - Baseline (T0) - 3 months (T1) - 6 months (T2)
**Maguire et al. (1983)**	Individual Face-to-face	**Physical/Psychosocial** Counselling/Education: - Nurse advised range of movement exercises for arm. - Encouragement to look at and discuss scar and loss of breast. - Demonstration of possible external breast protheses. - Home-visit post-discharge to assess upper limb monitor adherence to exercises and counselling. - Encouragement of return to work and social reintegration.	Nurse specialist	Throughout inpatient stay post-surgery (varied among participants). Followed up at home visit every two months until deemed fit for discharge.	None	***RTW:*** Yes/No/ Non-Applicable ***Response to scar, prosthesis and breast loss:*** Interview response (satisfied, neutral, dissatisfied) ***Perceived Impact on Swelling, Pain, and Disability:*** *Self report* ***Social adjustment:*** Single item question on problems with social adjustment ***Housework:*** Single item question on problems with housework ***Marital adjustment:*** ***Concurrent physical illness:*** *Measured at:* - 3 months - 12 months - 18 months
**Author**	**Intervention Characteristics**					
	**Format**	**Content**	**Facilitator(s)**	**Duration**	**Theoretical Framework**	**Outcome measures**
**Maunsell et al. (1996)**	Individual Face-to-face and Telephone	**Psychosocial:** Interventions included mix of information, education, support, counselling and referral where required.	Social worker	Telephone screening every 28 days for total of 12 screening calls.	Brief crisis intervention model.	***RTW:*** Binary Yes/No returned to work ***Working hours/week:*** Number of hours. ***Psychologic symptoms:*** General Health Questionnaire [GHQ] ***Psychologic distress:*** Psychiatric Symptom Index ***Social support:*** Social Support Questionnaire ***Stressful Life Events****:* Life Experiences Survey ***Marital satisfaction:*** The Locke-Wallace Marital Adjustment Test [LWMAT] ***Depression and Anxiety:*** Diagnostic Interview Schedule [DIS] ***Physical Health:*** Self-report **Outcomes measured:** Baseline (T0), 3 months (T1), 6 months (T2)
**Mourgues et al. (2014)**	Face-to-face Group	**Physical/Psychosocial** Multicomponent including physiotherapy, nutritional advice, thermal water treatment, daily two-hour physical activity, running and basic dietary follow-up. Consultation with dietitian every six months.	Physiotherapist, Dietitian,	15-day programme. Daily two-hour physical activity.	None	***Occupational activity:*** Total hourly volume of overall & occupational activity. ***Daily abilities:*** Perception whether health problems impacted on activities. **Outcomes measured:** Baseline, 6 & 12 months
**Rogers et al. (2009)**	Face-to-Face and home-based exercises | Group and Individual	**Physical/Psychosocial** The BEAT Cancer programme: • 12 individual supervised exercise • Home-based exercise • 3 individual face-to-face counselling sessions. • Six discussion group sessions addressing: Social support, Journaling, Time Management, Stress Management, Dealing with Exercise Barriers, Behaviour modification	Clinical Psychologist, Exercise specialists (certified by American College of Sports Medicine or certified eligible).	12-week programme.	Social Cognitive Theory	***Sick days:*** Self-report number of days off work ***Quality of life****:* Functional Assessment of Cancer Therapy—Breast (FACT-B) & FACT- G (General) ***Fatigue:*** FACT—Fatigue (FACT-F) ***Endocrine symptoms:*** FACT—Endocrine Symptoms (FACT-ES) ***Cognitive function:*** FACT—Cognitive ***Sleep dysfunction:*** Pittsburgh Sleep Quality Index (PSQI) ***Physical activity behaviour:*** The Godin Leisure-Time Exercise Questionnaire ***Motivational readiness for physical activity:*** Self-report of stage of change ***Lower extremity pain and function:*** Western Ontario and McMaster Universities Arthritis Index (WOMAC)

#### Format and delivery

All studies included face-to-face intervention delivery. Two studies also delivered the intervention partially by telephone [21, 28]. Three interventions including exercise involved home-based self-directed exercises [25, 26, 31]. Four interventions were group-based [29–32], four were individual [21, 25, 27, 28] and one intervention was blended (group and 1:1) [26]. Individual session length was not described in all papers, however, was usually indicated in physical interventions where session lengths varied between 60 and 120 min (Table 2) [30, 32].

#### Theoretical framework

Most studies (*n* = 6) did not report a specific theoretical framework/model used to guide intervention design or delivery (Table 2). Only three studies reported application of theoretical frameworks. These included the Biopsychosocial Model [21], the Brief Crisis Intervention Model [28], and Social Cognitive Theory [26].

### Comparator

All studies reported comparators of usual care. This most frequently included provision of written materials (e.g. physical activity [26, 30]; ‘Work and Cancer’ [21]). Usual care also included encouragement of healthy lifestyles [25], nurse support [31], a psychologic follow-up programme and physiotherapy [28], or dietitian consultation [32].

### Outcomes and outcome measures

All work outcomes were assessed by self-report (Table 2). The most assessed work outcome was sick leave/RTW (binary yes/no question if the participant had returned to work in some capacity). The second most commonly measured outcome was number of working hours, followed by number of sick days. One study assessed occupational (work) capacity by asking women if their health problems adversely impacted on ability to complete occupational activities [32]. The most frequently measured patient-reported outcomes included physical (*n* = 7) and psychological (*n* = 6) sequalae, and QoL (*n* = 4). Other outcomes included sleep, symptom burden, household tasks, social activities, and marital adjustment. Outcome measures varied across studies, with little overlap in most cases.

### Risk of bias within studies

**Fig. 2:**
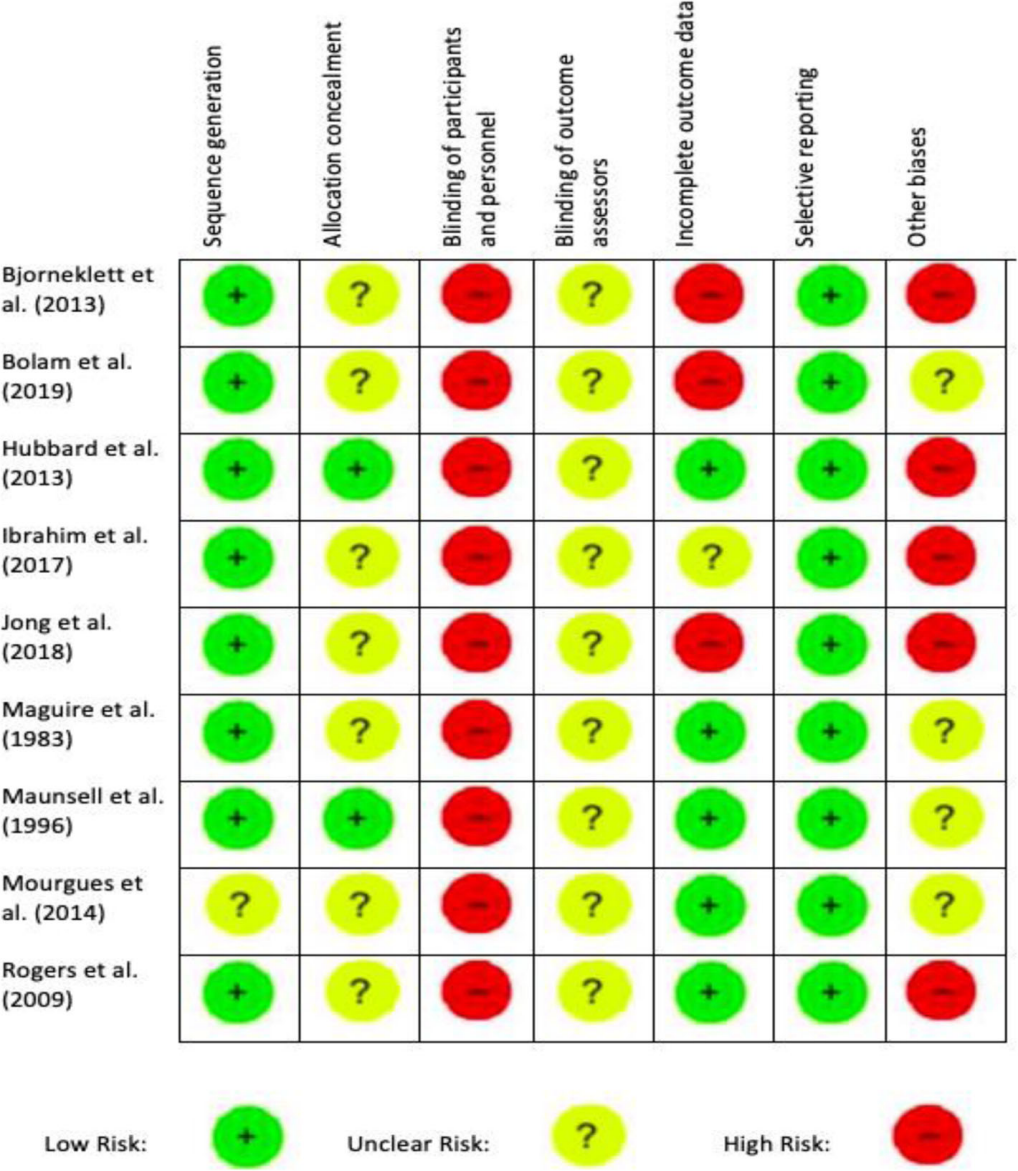
Risk of Bias Assessment

A risk of bias assessment summary for each included study is indicated in Fig. 2. Every study was deemed high risk for blinding of participants and personnel as due to the nature of the intervention it was not possible to blind participants. Participants assessed their own outcomes (as using self-reported questionnaires) and so it is unclear if awareness of their randomised allocation might have directly influenced the outcomes. For further details, see Supplementary Material 3.

### Synthesis of results

Meta-analysis was possible for a limited number of studies for work-related outcomes: number of sick days taken, still on sick leave (yes/no), and working hours. However, because the majority of interventions, and reported outcome measures varied, narrative synthesis was also conducted. A summary of results of individual studies can be found in Table 3.

**Table 3:** Results of individual studies included in the systematic review and meta-analysis

Author	Work outcomes			Other outcomes			
	Sick Leave / RTW (Y/N)	Working hours	Other:	Physical	Psychological	QoL	Other
**Björneklett et al. (2013)**	*(Sick Leave - Days):* No significant differences at 0, 2, 6, or 12 months.						*Healthcare utilisation:* Not significant re. visits to medical specialists, GPs or physiotherapists. However, women treated with chemotherapy in intervention group had significantly more visits with ‘Other’ healthcare professionals than the control at 6 and 12 months. *Health economics:* Intervention was significantly greater in cost compared to control.
**Bolam et al. (2019)**	*% of sick leave at that timepoint:* No significant differences between the two groups at 2 years.			*Cancer-related fatigue (CRF):* Significant differences between RT-HIIT and control groups for CRF and Cognitive CRF in favour of RT-HIIT who experienced improvements in both. *Physical symptoms (Item MSAS):* No significant differences	*Psychological symptoms (Item of MSAS):* No significant differences between groups. *Emotional functioning (item of EORTC):* No significant differences	*QoL:* No significant differences between the two groups.	*Total symptoms:* Sig. ↓ total symptoms than UC at 2 years in favour of AT-HIIT intervention. *Symptom burden:* Sig. ↓ symptom burden than UC at 2 years in favour of AT-HIIT intervention.
**Hubbard et al. (2013)**	*Number of days sick leave:* No significant differences		*Change in employment pattern:* No significant differences between groups	*Fatigue:* No significant differences *Physical functioning (item of FACT-B):* No significant differences	*Emotional functioning (item of FACT-B):* No significant differences	*QoL:* Significant differences between groups on breast cancer specific QoL in favour of intervention who experienced improvements.	
QoL: Quality of Life; RT-HIIT = Resistance Exercise and High-Intensity Interval Training; AT-HIIT = Moderate Intensity Aerobic Exercise and High-Intensity Interval Training							
**Author**	**Work outcomes**			**Other outcomes**			
	**Sick Leave / RTW (Y/N)**	**Working hours**	**Other:**	**Physical**	**Psychological**	**QoL**	**Other:**
**Ibrahim et al. (2017)**		Not reported for control group therefore unable to ascertain if significant.		*Upper limb function:* No significant differences			
**Jong et al. (2018)**	*Return to work (Y/N):* No significant difference between groups			*Fatigue:* No significant differences *Confidence in fatigue reduction:* Significantly more confident in fatigue reduction in favour of intervention group. *Adequate relief of fatigue (Y/N):* Significantly more relief of fatigue in favour of intervention group.	*Psychological distress:* No significant differences in levels of anxiety Significantly less depressive symptoms at 3 months in favour of intervention. *Emotional functioning (item of EORTC):* No significant differences	*QoL:* Significantly less nausea and vomiting at six months in favour of Intervention group. No significant differences for other outcomes.	*Impact of events:* No significant differences. *Treatment expectations:* Intervention group had significantly higher treatment expectations compared to control.
**Maguire et al. (1983)**	*RTW (Y/N):* No significant differences			*Upper limb swelling, pain and disability* No significant differences	*Reaction to scar, prosthesis and breast loss:* Intervention group were significantly more satisfied with scar, prothesis, breast loss, compared to control.		*Housework, Social adjustment, Martial adjustment:* No significant differences between groups.
QoL: Quality of Life; RT-HIIT = Resistance Exercise and High-Intensity Interval Training; AT-HIIT = Moderate Intensity Aerobic Exercise and High-Intensity Interval Training							
**Work outcomes**				**Other outcomes**			
**Author**	**Sick Leave / RTW (Y/N)**	**Working hours**	**Other:**	**Physical**	**Psychological**	**QoL**	**Other:**
**Maunsell et al. (1996)**	*RTW (Y/N):* No significant differences	*Working hours (per week):* No significant differences		*Physical health:* No significant differences	*Psychological distress:* No significant differences		*Perception of health. Functional status, Social activity, Marital relations:* No significant differences
**Mourgues et al. (2014)**			*Occupational activity (Work):* Significant improvement in ability to perform work activities at 12 months in favour of intervention group.				*Overall activities:* Significant differences between groups in favour of intervention who had increased resumption of overall activities in first 12-month period. *Non- occupational activity (Family, household tasks and volunteerism):* Significant improvement in ability to perform family activities at 12 months, in favour of intervention. *Cost-effectiveness:* Significant differences at 12 months, in favour of intervention
**Rogers et al. (2009)**	*Number of sick days in past month:* No significant differences			*Fatigue, Joint pain, Physical function:* No significant differences *Physical functioning (item of FACT-B):* No significant differences *Joint stiffness:* Significantly greater perceived joint stiffness in intervention group compared to control.	*Emotional functioning (item of FACT-B):* No significant differences	*QoL:* Significant improvement in social well-being in favour of intervention Not significant for other QoL outcomes	*Cognition, Perceived health, Endocrine symptoms, Sleep dysfunction:* No significant differences

QoL: Quality of Life; RT-HIIT = Resistance Exercise and High-Intensity Interval Training; AT-HIIT = Moderate Intensity Aerobic Exercise and High-Intensity Interval Training

#### Effectiveness of interventions on work outcomes - meta-analysis

Limited meta-analysis was possible on work outcomes including number of sick days taken (at six and 12-months) [21, 29], if someone remained on sick leave (at 12 months) [28, 29], and the number of working hours (at 12 months) [28, 32].

**Fig. 3:**
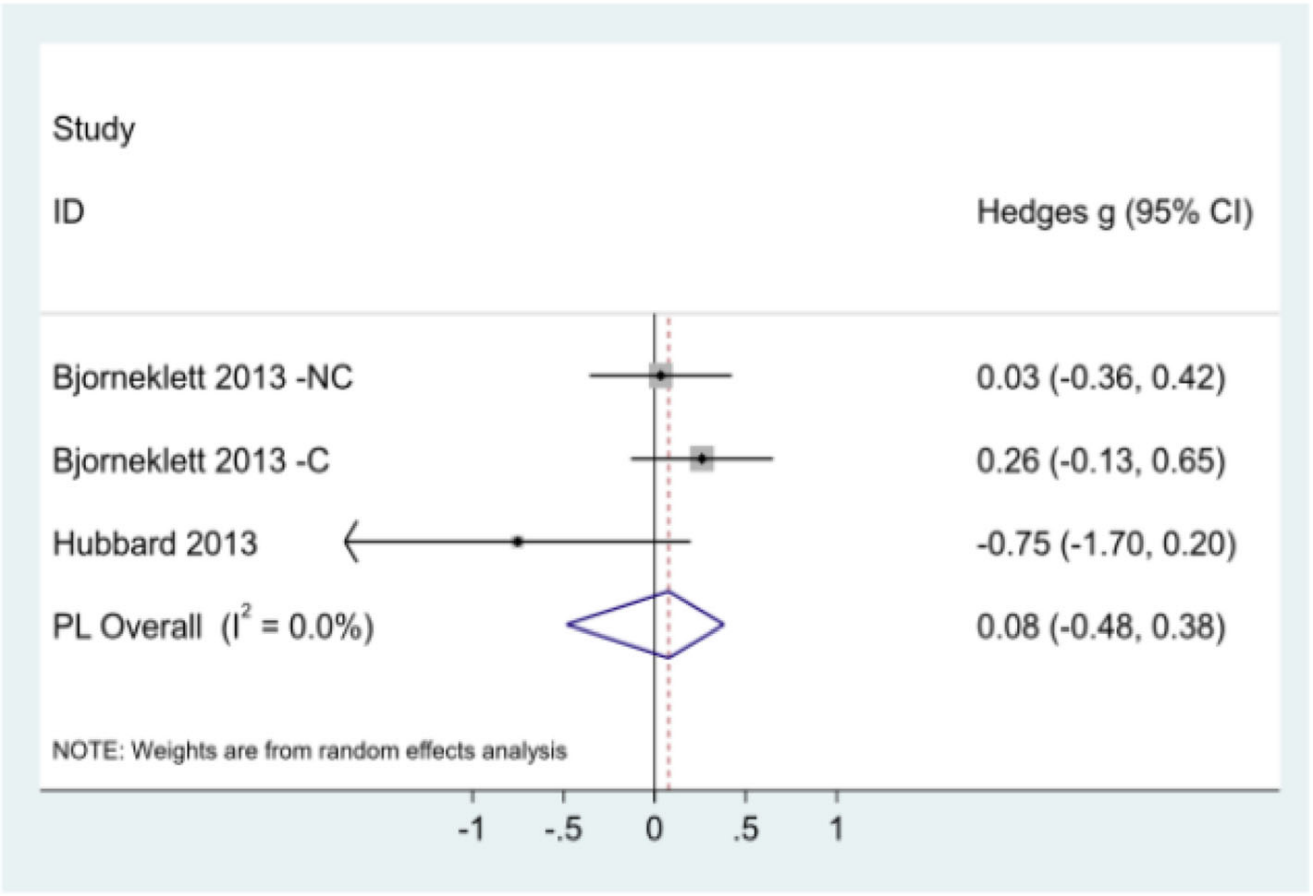
Meta-analysis of number of sick days taken at six months. NC: Non-Chemotherapy Group; C: Chemotherapy Group

**Fig. 4:**
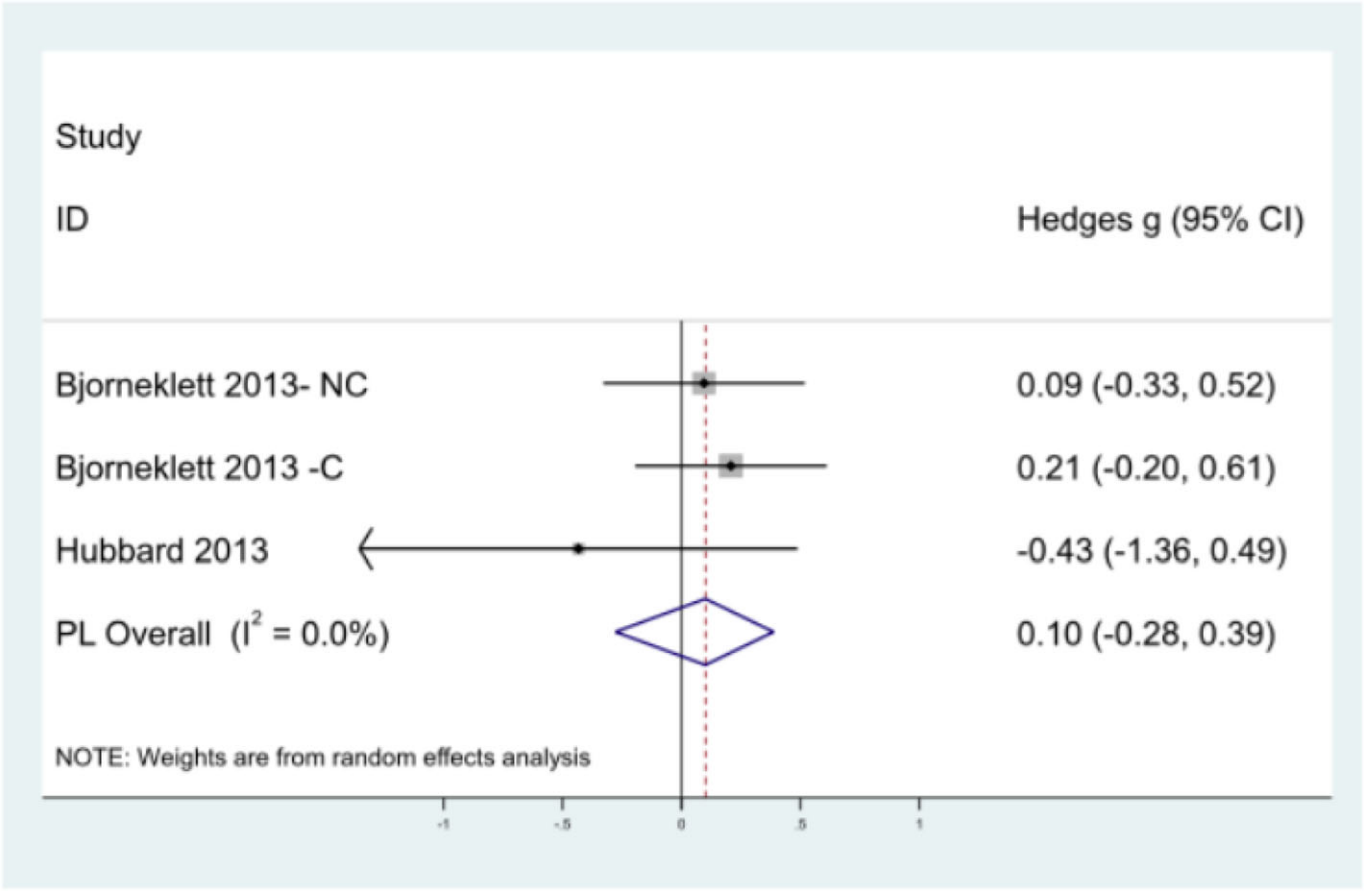
Meta-analysis of number of sick days taken at twelve months. NC: Non-Chemotherapy Group; C: Chemotherapy Group

Number of sick days taken (six months and 12 months): Data for the number of sick days taken were available for two studies at six and 12 months [21, 29]. A random effects model was used due to heterogeneity between studies. At six months (Fig. 3), pooled analysis resulted in a non-significant overall effect of − 0.08 (95% CI: − 0.48, 0.38). Björneklett et al. observed an effect close to zero of 0.03 (Hedge’s *g*) between non-chemotherapy intervention and control groups (95% CI: − 0.36, 0.42), and a small effect size of 0.26 (Hedge’s *g*) between chemotherapy intervention and control groups (95% CI: − 0.13, 0.65) [29]. Hubbard et al. (2013) [21] observed a small effect size of − 0.75 between intervention and control groups (CI: − 1.70, 0.20). Figure 4 provides the results from meta-analysis of outcomes at twelve months. Björneklett et al. observed an effect close to zero of 0.09 (Hedge’s *g*) between non-chemotherapy intervention and control groups (CI: − 0.33, 0.52) and a small effect size of 0.21 (Hedge’s *g*) between chemotherapy intervention and control groups (95% CI: − 0.20, 0.61) [29]. Hubbard et al. observed a small effect size of − 0.43 between intervention and control groups (CI: − 1.36, 0.49) [21]. Pooled analysis indicated a non-significant overall very small effect of 0.10 (CI: − 0.28, 0.39).

#### Sick leave (Y/N) at 12 months - meta-analysis

**Fig. 5:**
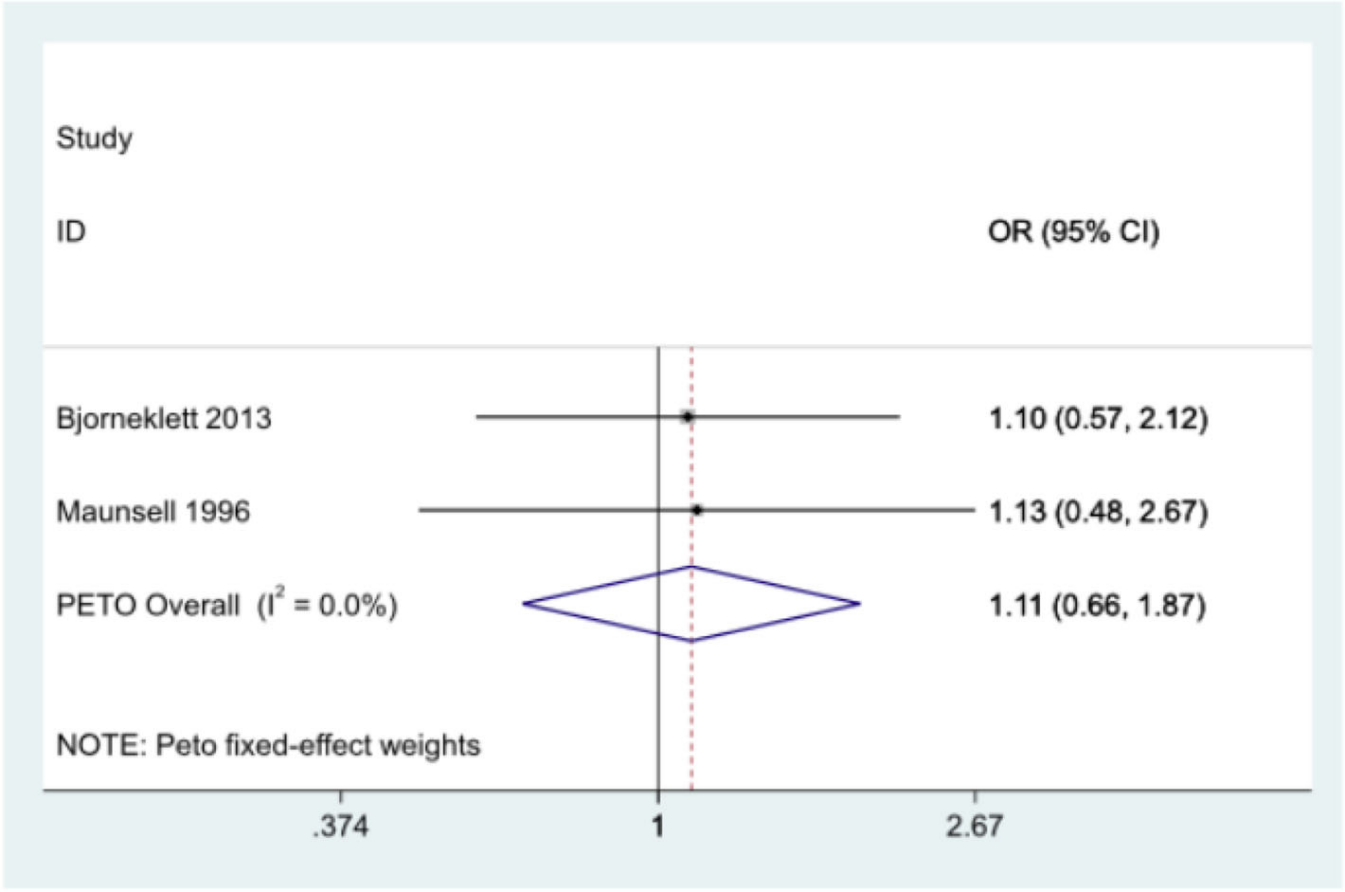
Meta-analysis of sick leave (Yes/No) at twelve months

Sick leave data were available for two studies at 12 months [28, 29], however results were not statistically significant between intervention and control groups (Fig. 5). Björneklett et al. observed an OR of 1.10 (95% CI: 0.57, 2.12) for the association of any (vs no) sick leave between intervention vs control groups whereas Maunsell et al. observed an OR of 1.13 (95% CI: 0.48, 2.68) for the associations of any sick leave between the intervention and control groups [28, 29]. Pooled analysis resulted in an overall OR (peto) of 1.11 (95% CI: 0.66, 1.87), which was close to 1.

#### Working hours at 12 months - meta-analysis

**Fig. 6:**
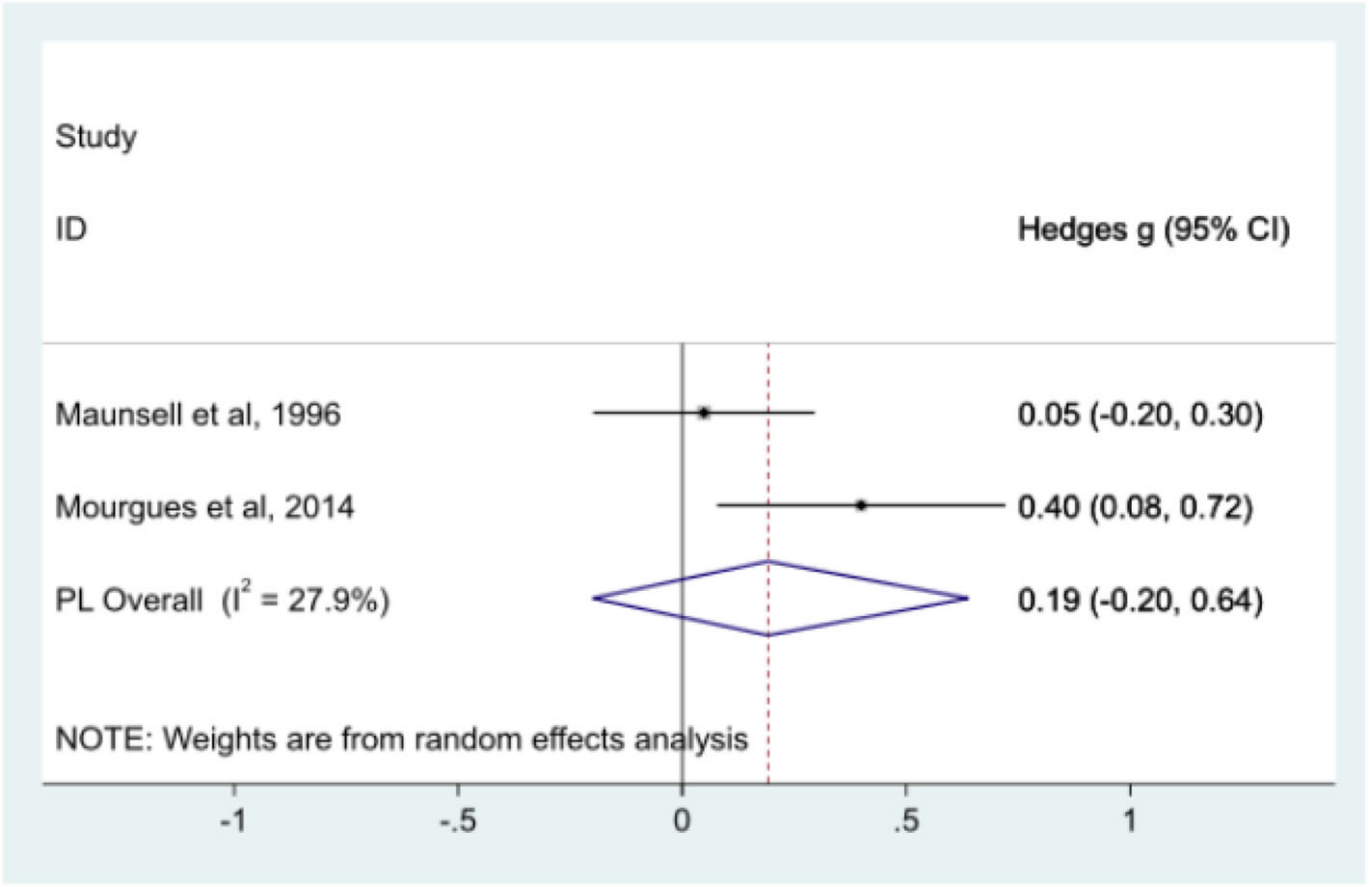
Meta-analysis of working hours at twelve months

Working hours data were available for two studies at 12 months however, there was no significant evidence of a difference between the control and intervention groups (Fig. 6) [28, 32]. Maunsell et al. observed an effect size close to zero of 0.05 (Hedge’s *g*) between intervention and control groups (95% CI: − 0.20, 0.30) whereas Mourgues et al. observed a small-medium effect size of 0.4 (Hedge’s *g*) between groups (95% CI: 0.08, 0.72) [28, 32]. Pooled analysis indicated an overall small effect of 0.19 (95% CI: − 0.20, 0.64). Heterogeneity measures indicated a I^2^ value of 28.27 and H^2^ value of 1.39. A random effects model for pooling analysis is shown in Fig. 6.

##### Effectiveness of interventions on work outcomes - narrative synthesis

Of the nine included studies, only one study reported statistically significant differences in favour of the intervention group for increased ‘occupational activity’ [32]. Findings indicated that the intervention group had significantly higher ability to perform work activities at 12-months compared to the control group. The remaining eight studies did not report any statistically significant differences between groups, however increased numbers of the intervention group returning to work. or taking less sick leave compared to usual care, were reported in three studies; (i) the intervention group in Hubbard et al. [21] study reported 53 fewer sick leave days compared to the control, (ii) Jong et al., [31] found 53% of the intervention group did RTW at six months compared to 23% of the control, and (iii) 76% of intervention group returned to work compared to 54% of the control in the study by Maguire et al., [27].

##### Effectiveness of interventions on other health outcomes - narrative synthesis

Other health outcomes were considered secondary outcomes in this review (Table 3).

Of the seven studies reporting physical outcomes [21, 25–28, 30, 31], three reported statistically significant differences, which were not always positive. For example, Rogers et al. observed greater perceived joint stiffness in the intervention group compared to the control [26]. Four studies measured fatigue [21, 26, 30, 31], two of which reported statistically significant differences in favour of the intervention group [30, 31]. While effect sizes in both studies were small, Bolam et al. reported statistically significant differences between the RT-HIIT intervention and control groups in total cancer-related fatigue (CRF) and in Cognitive CRF [30]. While there were no statistically significant differences between groups in Multidimensional Fatigue Inventory (MFI) and Fatigue Quality List (FQL) scores in the Jong et al. study, they did report a statistically significantly higher percentage of women in the intervention (51%) experiencing fatigue reduction compared to the control (19%) at 3-months [31].

Six studies reported psychological outcomes [21, 26–28, 30, 31], only two of which demonstrated statistically significant results [27, 31]. Jong et al. reported significant differences for the intervention group in depression at the three-month time point [31]. Maguire et al. reported that participants in the control were statistically significantly more dissatisfied with scarring and prosthesis, than the intervention group [27].

Three out of four studies reported enhanced overall QoL, or components of QoL outcomes in favour of the intervention group [21, 26, 31]. While there were no statistically significant differences between groups in the total scores of the EORTC-QLQ-C30, Hubbard et al. identified statistically significant differences between groups on the Breast Cancer Subscale at six-months, in favour of the intervention group [21]. Jong et al. identified a statistically significant improvement in nausea and vomiting (an EORTC-QLQ-C30 item) for the intervention group at six-months [31]. Finally, Rogers et al. observed statistically significant improvements in social wellbeing for the intervention group compared to control, with a large effect size of 0.76 [26].

##### Cost-effectiveness - narrative synthesis

Only two studies measured cost-effectiveness [29, 32]. Morgues et al., reported cost-effectiveness for the intervention at 12 months [32]. They examined direct (e.g., consultations, transport, thermal treatment) and indirect costs (e.g., out-of-pocket expenses associated with disease) in their cost effectiveness analysis (CEA). In contrast, Björneklett et al. concluded that costs to society were not reduced with the intervention in its present form [29]. They reported total costs for the intervention group (cost of sick leave and consumption of health services) were higher at all time-points, reaching statistical significance between groups at 12 months. When adding the cost of the intervention (€2300) in addition to the costs of sick leave and healthcare utilisation, the costs for the intervention group were significantly higher at all time-points. While there were no significant differences between groups regarding visits to medical specialists, GPs or physiotherapists, women treated with chemotherapy in the intervention group had significantly more visits with ‘other’ healthcare professionals than the control group at 6 and 12 months.

## Discussion

The objective of this review was to determine efficacy of rehabilitation interventions on work outcomes and identify core content and suitable measurement tools for interventions for women with breast cancer. The findings highlight variability across interventions for women with breast cancer, in intervention effectiveness, content, and delivery, currently available in published literature. Therefore, it is challenging to offer definitive recommendations on what constitutes an effective intervention to support work outcomes for women with breast cancer.

Only one study observed statistically significant differences in work outcomes between intervention, and control groups, observing greater resumption of work and participation in overall work activities at 12-months [32]. The success of this study could be partially explained by its multidisciplinary format providing exercise, psychological and dietary advice or to the sample size which may have been more adequately powered than other studies. A recent Cochrane review identified moderate quality evidence for multidisciplinary interventions in enhancing RTW rates across all cancer types, underlining the potential effects of a multicomponent rehabilitative approach [15]. Despite this, some aspects of the intervention (e.g., thermal water treatment) may be impractical if applied to informing a work-focused intervention, where thermal water treatment facilities are not widely available in healthcare services. In addition, no work-related content was included in the intervention. Lack of statistically significant impact on work outcomes across the other studies can perhaps be explained by the fact that the majority of interventions did not specifically focus on work in their interventions. Evidence suggests that interventions which are designed to target management of a specific concern, result in significant effects on that specified outcome [33]. While three studies in this current review included work components in their intervention, the content varied, and no statistically significant results were observed for work outcomes [21, 27, 29]. This could be because there was insufficient work-specific content in the interventions. Another explanation could be that the studies comprised of small sample sizes. For example, despite Hubbard et al. including work-specific content in their intervention, only 18 women participated [21]. Future RCTs with larger samples may provide further insight into effectiveness using work-directed approaches. While work outcomes were measured across all studies by self-report, they varied from quantifying number of working days/hours to whether the participant had returned to work (yes/no response). Measuring RTW by binary yes/no could be problematic where the definition of RTW is blurred. As Lamore et al. highlighted, RTW does not necessarily indicate that a previous lifestyle is completely restored, and there needs to be clarity as to the definition of RTW [34]. Researchers could consider if work outcomes imply RTW full-time or part-time, and perhaps perceived satisfaction with the outcome.

It is well documented that treatment and disease-related symptoms such as cancer-related fatigue, cognitive changes, and anxiety can impact on work ability and could be targeted as part of a RTW intervention [9, 10]. Therefore, physical, psychological and QoL outcomes were also examined in this review. Outcomes differed widely across studies, with varying results making it challenging to offer definitive recommendations for the content and delivery of interventions to support return to work. Of the four studies measuring fatigue, significant improvements were observed only in a physical intervention [30]. Interventions which deliver aerobic exercise have previously been cited in a Cochrane Review as beneficial in reducing cancer-related fatigue [35]. Another Cochrane Review reported limited evidence for psychosocial interventions in reducing fatigue unless specifically targeting fatigue [36]. An update of evidence is warranted however as the review was conducted more than a decade ago. In contrast, of the four studies measuring the impact of interventions on QoL, three which reported improvements, delivered both physical and psychosocial interventions. This underlines the importance of a multidisciplinary approach in RTW interventions in targeting a holistic range of treatment- and disease-related factors that impact on work for women with breast cancer. Interventions targeting QoL have varied considerably in participants, delivery and content making it difficult to arrive at a firm conclusion regarding effectiveness, although a Cochrane review tentatively concluded potential benefit of interventions which are educational and offer supportive attention [37]. Some specific outcomes of interest that are known to impact on work, were under-reported. For example, financial status, social support and cognitive dysfunction were less commonly reported outcomes, but could be considered, particularly as they can impact on RTW [8, 38]. In addition, considering upper limb function could be important for women with breast cancer, who are more likely to experience upper limb impairment compared to other cancer groups [8]. Lymphoedema, for example, is known to compound RTW challenges where there may be restrictions in mobility or heavy lifting, for example [39]. A multicomponent approach in rehabilitation may help to address wide-ranging disease and treatment-related side-effects that impact on RTW [40–42].

There also remain few studies reporting intervention cost-effectiveness. This gap is important to note as economic evaluation is a key consideration for decision-makers and is also outlined as a pillar for evaluation of complex interventions under the Medical Research Council framework for complex interventions [23]. Two of the nine studies reporting cost-effectiveness, observed contrasting results. One study observed higher costs for the intervention group who typically sought greater use of healthcare services than the control group [29]. This could be because women in the intervention received education on availability of healthcare professionals to assist with symptom management. Greater self-awareness of one’s own health status could lead to a willingness to self-manage health and seek out appropriate health services. This could lead to reduced or self-managed co-morbidity in the future which could provide a cost-benefit for the intervention. In contrast, Mourgues et al. observed enhanced work outcomes, and reported the intervention was cost-effective at 12 months [32]. It is not clear however if, like Björneklett et al., consultations with healthcare professionals other than medical professionals were also included in the analysis [29]. Mourgues et al. did however use two facilitators as part of their intervention, whereas Björneklett et al. use seven from a variety of disciplines [29, 32]. This is likely to have impacted on the overall costs of each intervention. While multidisciplinary interventions have been identified as impacting on RTW rates in cancer care [15], researchers should take into consideration the overall cost impact if including a large range of disciplines. Future study designs could factor in healthcare utilisation into CEA both in the short- and long-term and avoid small sample sizes which are considered a limitation for calculating CEA.

On reviewing health behaviour change theory underlying study interventions, no clear conclusions on a preferred or most effective model can be drawn. Of the nine studies, only three reported using a theoretical framework, all of which varied. This gap has been previously echoed for other rehabilitation interventions for those with cancer [8, 34, 43] and is noteworthy as incorporating insights from theory is recommended as a key consideration when developing complex interventions [23]. In this current review, none of the theories reported in the three studies were specific to work rehabilitation. For example, Social Cognitive Theory [44] which is often used in behaviour change interventions, was reported in one study [26]. This theory holds promise for understanding RTW motivations, expectations of efficacy, and predicting one’s ability to achieve desired outcomes (i.e., work outcomes), but can be vague in operationalisation [45]. Similarly, while the Biopsychosocial model reported by Hubbard et al. is holistic in nature considering biological, psychological and social factors, its generic nature can limit its direct application to work rehabilitation research and practice [21, 45, 46]. With this in mind, the evidence base beyond this current review can be explored for more specific models to occupational rehabilitation. A Cancer and Work Model was developed by Feuerstein et al., for all cancer cohorts, it includes factors that can be addressed by healthcare professionals, individuals living with or beyond cancer, and employers, and could be considered in intervention development [47].

This review provides an update on previous literature exploring return to work interventions for women with breast cancer where only one of four studies included in that review was controlled [19]. In contrast, all nine studies in this review were RCTs, potentially reducing selection bias. This is a promising indication that more rigorous methods are being employed in intervention evaluation. Most studies (*n* = 6) in this current review were published since 2010 indicating growing research in recent years. Furthermore, a number of protocols for upcoming RCTs testing work interventions for women with breast cancer have been published [48–50], and it is likely that there will be an increased evidence-base to further explore feasibility and effectiveness in the future. There are however limitations in intervention development, where there is a lack of pilot and feasibility studies, which are advocated by several models for guiding intervention development [23, 51]. Three of the nine studies in this current review were pilots, and the six remaining RCT studies did not report a pilot study prior to the full trial. While recruitment, adherence and attendance rates were referred to briefly in four studies [25, 28–30], feasibility was only explicitly reported in two [21, 26]. Lack of piloting and feasibility research can lead to methodological challenges. For example, Jong et al. [31] did not report any pilots or feasibility testing of their intervention and experienced recruitment issues during the RCT. Despite adding an additional recruitment site, recruitment remained challenging.

### Strengths and limitations

This review offers a collective insight into current evidence available on interventions for women with breast cancer that impact on work outcomes. A systematic search process was applied, limiting bias, and meta-analysis was possible for a number of outcomes which offers a statistical measure of the impact of intervention. Backwards and forwards chaining was completed on relevant texts to ensure complete inclusion of studies. Limitations were also identified. For practical reasons, a limit was applied to eligibility criteria for English-text only. However, this may have restricted other potential texts from being included in the final review. Meta-analysis was completed where possible, however, it is acknowledged that results need to be taken with caution as only two studies could be pooled for each analysis and the interventions examined might have been too heterogenous. There are numerous arguments for and against the meta-analysis of a small number of studies. Valentine and colleagues (2010) [52] argue, however, that given the need for a conclusion, two studies can be used for meta-analysis as all other synthesis techniques are less transparent. The study sizes were also small in several of the included studies, which may have limited the reliability and strength of evidence (power) to support the effectiveness of the interventions being evaluated.

### Recommendations for future practice and research

In the absence of a sufficient evidence-base and the ability to make definitive recommendations, clinicians could consider multidisciplinary interventions to support women with breast cancer to return to work, as advocated by de Boer et al. [15]. While rehabilitation interventions including work components did not observe statistically significant results, the value of work components cannot be ruled out, particularly where the only study to use a work-directed approach (e.g., work accommodations and modifications) was underpowered. Further research in developing and evaluating RTW interventions for women with breast cancer is warranted. Despite enhanced rigour in the study-designs over the past decade, there remains a paucity in piloting and testing feasibility of work-specific interventions. Future research could incorporate a model of intervention development into the study-design. Patient-reported work outcomes have typically been reported in studies. Objective measures (exploring work performance, for example) could also be considered in future designs. Furthermore, sufficient sample sizes to ensure an adequately powered study are necessary.

## Conclusion

Interventions to support women with breast cancer to return to, or remain at, work remain scarce. Of the interventions that do exist, variability in content, and lack of evidence of the effectiveness on work outcomes, make it challenging to offer definitive recommendations for delivery of work-focused interventions. Despite this, studies of higher quality have emerged in the past decade with promising potential for an expanded evidence-base in the future. Future research in developing and evaluating work-focused interventions for women with breast cancer is warranted.

## Supplementary Information

**Additional file 1.**

**Additional file 2.**

**Additional file 3.**

## Data Availability

The datasets used and/or analysed during the current study available from the corresponding author on reasonable request.
