# Supplementary material for: Rehabilitation interventions to support return to work for women with breast cancer: a systematic review and meta-analysis
Source: BMC Cancer. 2021 Aug 5;21:895. doi: 10.1186/s12885-021-08613-x (PMC8340442; doi:10.1186/s12885-021-08613-x)
Supplement: Supplementary file 1 — Additional file 1. [file 12885_2021_8613_MOESM1_ESM.docx]

**SUPPLEMENTARY MATERIAL 1: Search Strategies**

**EMBASE**

1. 'breast cancer'/exp
2. (Breast NEAR/3 (cancer OR carcinoma* OR tumo?r* OR neoplasm*)):ti,ab
3. #1 OR #2
4. 'cancer survivor'/exp OR 'cancer survival'/exp
5. Survivor*:ti,ab
6. #4 OR #5
7. 'employability'/exp OR 'career'/exp OR 'career mobility'/exp OR 'career planning'/exp OR 'employment'/exp OR 'job change'/exp OR 'job characteristics'/exp OR 'job finding'/exp OR 'presenteeism'/exp OR 'absenteeism'/exp OR 'work capacity'/exp OR 'job accommodation'/exp OR 'job security'/exp OR 'work'/de
8. ((Work OR job OR employment) NEAR/3 (retention OR return OR retain OR cessation OR continuation OR maintain* OR opportunit* OR quit* OR loss OR non-retention OR resumption OR resume*)):ti,ab
9. (unemploy* OR career* OR employability OR demotion OR absenteeism OR presenteesism OR underemployment OR productivity OR reemployment or re-employment OR ‘sickness absence’ OR “sick leave”):ti,ab
10. ((work*) Near/3 (Stay* OR remain* OR continu* OR adjustment* OR readiness OR functioning OR limitation* OR participation OR transition* OR capacity OR status)):ti,ab
11. (Workplace Near/3 (intervention* OR discrimination OR accommodation* OR support*)):ti,ab
12. #7 OR #8 OR #9 OR #10 OR #11
13. #3 AND #6 AND #12

**Medline (OVID)**

1. exp Breast Neoplasms/
2. (Breast adj3 (cancer OR carcinoma* OR tumo?r* OR neoplasm*)).ti,ab.
3. or/1-2
4. Survivors/ OR Cancer Survivors/
5. Survivo?r*.ti,ab.
6. or/4-5
7. exp work/ or return to work/ or exp employment/ or unemployment/ or exp occupations/ or workplace/ or Rehabilitation, Vocational/ or Sick Leave/ or Absenteeism/ or Presenteeism/ or Retirement/ or Workers' Compensation/ or Job Satisfaction/ or Employee Grievances/ or Workload/ or Personnel Turnover/ or Work Engagement/ or Work Performance/ or exp Occupational Stress/
8. ((Work OR job OR employment) adj3 (retention OR return OR retain OR cessation OR continuation OR maintain* OR opportunit* OR quit* OR loss OR non-retention OR resumption OR resume*)).ti,ab.
9. (unemploy* OR career* OR employability OR demotion OR absenteeism OR presenteesism OR underemployment OR productivity OR reemployment or re-employment OR sickness absence OR sick leave).ti,ab.
10. ((work*) adj3 (Stay* OR remain* OR continu* OR adjustment* OR readiness OR functioning OR limitation* OR participation OR transition* OR capacity OR status)).ti,ab.
11. (Workplace adj3 (intervention* OR discrimination OR accommodation* OR support*)).ti,ab.
12. or/7-11
13. and/3,6,12

**CINAHL**

1. (MH "Breast Neoplasms+")
2. TI (Breast N6 (cancer* OR neoplasm* OR carcinoma* OR tumo?r*)) OR AB (Breast N6 (cancer* OR neoplasm* OR carcinoma* OR tumo?r*))
3. S1 OR S2
4. (MH "Cancer Survivors") OR (MH "Survivors")
5. TI (survivor*) OR AB (survivor*)
6. S4 OR S5
7. (MH "Employment+") OR (MH "Career Mobility") OR (MH "Promotion and Tenure") OR (MH "Occupations and Professions") OR (MH "Income") OR (MH "Unemployment") OR (MH "Job Satisfaction+") OR (MH "Absenteeism") OR (MH "Adaptation, Occupational") OR (MH "Stress, Occupational") OR (MH "Presenteeism") OR (MH "Productivity") OR (MH "Professional Image") OR (MH "Professional Recognition") OR (MH "Work-Life Balance") OR (MH "Time Management") OR (MH "Work Capacity Evaluation") OR (MH "Work") OR (MH "Work Engagement")
8. TI ((Work OR job OR employment) N3 (retention OR return OR retain OR cessation OR continuation OR maintain* OR opportunit* OR quit* OR loss OR non-retention OR resumption OR resume*)) OR AB((Work OR job OR employment) N3 (retention OR return OR retain OR cessation OR continuation OR maintain* OR opportunit* OR quit* OR loss OR non-retention OR resumption OR resume*))
9. TI (unemploy* OR career* OR employability OR demotion OR absenteeism OR presenteesism OR underemployment OR productivity OR reemployment or re-employment OR “sickness absence” OR “sick leave”) OR AB (unemploy* OR career* OR employability OR demotion OR absenteeism OR presenteesism OR underemployment OR productivity OR reemployment or re-employment OR “sickness absence” OR “sick leave”)
10. TI ((work*) N3 (Stay* OR remain* OR continu* OR adjustment* OR readiness OR functioning OR limitation* OR participation OR transition* OR capacity OR status)) OR AB ((work*) N3 (Stay* OR remain* OR continu* OR adjustment* OR readiness OR functioning OR limitation* OR participation OR transition* OR capacity OR status))
11. TI (Workplace N3 (intervention* OR discrimination OR accommodation* OR support*)) OR AB (Workplace N3 (intervention* OR discrimination OR accommodation* OR support*))
12. S7 OR S8 OR S9 OR S10 OR S11
13. S3 AND S6 AND S12

**PsycINFO**

1. DE "Breast Neoplasms"
2. TI (Breast N6 (cancer* OR neoplasm* OR carcinoma* OR tumo?r*)) OR AB (Breast N6 (cancer* OR neoplasm* OR carcinoma* OR tumo?r*))
3. S1 OR S2
4. DE "Survivors"
5. TI (survivor*) OR AB (survivor*)
6. S4 OR S5
7. DE "Social Support" OR DE "Employee Productivity" OR DE "Occupational Health" OR DE "Reemployment" OR DE "Supervisor Employee Interaction" OR DE "Employment Status" AND DE "Working Conditions" OR DE "Working Women" OR DE "Workplace Intervention" OR DE "Unemployment" OR DE "Lower Income Level" OR DE "Career Development" OR DE "Working Women" OR DE "Occupational Stress" AND DE "Employee Absenteeism" OR DE "Employee Efficiency" OR DE "Employee Engagement" OR DE "Employee Layoffs" OR DE "Employee Motivation" OR DE "Employee Retention"
8. TI ((Work OR job OR employment) N3 (retention OR return OR retain OR cessation OR continuation OR maintain* OR opportunit* OR quit* OR loss OR non-retention OR resumption OR resume*)) OR AB((Work OR job OR employment) N3 (retention OR return OR retain OR cessation OR continuation OR maintain* OR opportunit* OR quit* OR loss OR non-retention OR resumption OR resume*))
9. TI (unemploy* OR career* OR employability OR demotion OR absenteeism OR presenteesism OR underemployment OR productivity OR reemployment or re-employment OR “sickness absence” OR “sick leave”) OR AB (unemploy* OR career* OR employability OR demotion OR absenteeism OR presenteesism OR underemployment OR productivity OR reemployment or re-employment OR “sickness absence” OR “sick leave”)
10. TI ((work*) N3 (Stay* OR remain* OR continu* OR adjustment* OR readiness OR functioning OR limitation* OR participation OR transition* OR capacity OR status)) OR AB ((work*) N3 (Stay* OR remain* OR continu* OR adjustment* OR readiness OR functioning OR limitation* OR participation OR transition* OR capacity OR status))
11. TI (Workplace N3 (intervention* OR discrimination OR accommodation* OR support*)) OR AB (Workplace N3 (intervention* OR discrimination OR accommodation* OR support*))
12. S7 OR S8 OR S9 OR S10 OR S11
13. S3 AND S6 AND S12

**Web of Science**

TS=((Breast NEAR/3 (cancer OR carcinoma* OR tumo?r* OR neoplasm*)) AND survivor* AND (((Work OR job OR employment) NEAR/3 (retention OR return OR retain OR cessation OR continuation OR maintain* OR opportunit* OR quit* OR loss OR non-retention OR resumption OR resume*)) OR (unemploy* OR career* OR employability OR demotion OR absenteeism OR presenteesism OR underemployment OR productivity OR reemployment or re-employment OR “sickness absence” OR “sick leave”) OR ((work*) Near/3 (Stay* OR remain* OR continu* OR adjustment* OR readiness OR functioning OR limitation* OR participation OR transition* OR capacity OR status)) OR (Workplace Near/3 (intervention* OR discrimination OR accommodation* OR support*))))

**Cochrane Library**

1. [mh “Breast Neoplasms“]
2. (Breast NEAR/3 (cancer OR carcinoma* OR tumor* OR tumour* OR neoplasm*)):ti,ab,kw
3. #1 OR #2
4. [mh “Survivors”] OR [mh “Cancer Survivors”]
5. (Survivor*):ti,ab,kw
6. #4 OR #5
7. [mh “work”] or [mh “return to work”] or [mh “employment”] or [mh “unemployment”] or [mh “occupations”] or [mh “workplace”] or [mh “Rehabilitation, Vocational”] or [mh “Sick Leave”] or [mh “Absenteeism”] or [mh “Presenteeism”] or [mh “Retirement”] or [mh “Workers' Compensation”] or [mh “Job Satisfaction”] or [mh “Employee Grievances”] or [mh “Workload”] or [mh “Personnel Turnover”] or [mh “Work Engagement”] or [mh “Work Performance”] or [mh “Occupational Stress”]
8. ((Work OR job OR employment) NEAR/3 (retention OR return OR retain OR cessation OR continuation OR maintain* OR opportunit* OR quit* OR loss OR non-retention OR resumption OR resume*)):ti,ab,kw
9. (unemploy* OR career* OR employability OR demotion OR absenteeism OR presenteesism OR underemployment OR productivity OR reemployment or re-employment OR “sickness absence” OR “sick leave”):ti,ab,kw
10. ((work*) Near/3 (Stay* OR remain* OR continu* OR adjustment* OR readiness OR functioning OR limitation* OR participation OR transition* OR capacity OR status)):ti,ab,kw
11. (Workplace Near/3 (intervention* OR discrimination OR accommodation* OR support*)):ti,ab,kw
12. #7 OR #8 OR #9 OR #10 OR #11
13. #3 AND #6 AND #12
