# Supplementary material for: Rehabilitation interventions to support return to work for women with breast cancer: a systematic review and meta-analysis
Source: BMC Cancer. 2021 Aug 5;21:895. doi: 10.1186/s12885-021-08613-x (PMC8340442; doi:10.1186/s12885-021-08613-x)
Supplement: Supplementary file 2 — Additional file 2. [file 12885_2021_8613_MOESM2_ESM.docx]

**SUPPLEMENTARY MATERIAL 2: Papers excluded from full-text review with reasons for exclusion**

| **Author** | **Reason for Exclusion** |
| --- | --- |
| Alsobrooks et al. (2010) [26] | No work-related outcomes |
| Cimprich et al, (2005) [27] | No work-related outcomes |
| Damkjaer et al. (2011) [28] | Study design other than RCT or quasi-experimental design (with comparator). Cohort design. |
| Désiron (2010) [29] | No clear reporting of work outcomes |
| Dietrich et al. (2016) [30] | No work-related outcomes |
| Hegel et al. (2011) [31] | No work-related outcomes |
| Hershman (2013) [32] | No work-related outcomes |
| Hoving, et al. (2009) [20] | Study design other than RCT or quasi-experimental design (with comparator). Systematic Review. |
| Khan et al. (2012) [33] | No work-related outcomes |
| Loh et al. (2013) [34] | No work-related outcomes |
| Lyons et al. (2015) [35] | No work-related outcomes |
| Meneses et al. (2007) [36] | No work-related outcomes |
| Meneses et al. (2009) [37] | No work-related outcomes |
| Meneses et al. (2020) [38] | No work-related outcomes |
| Newman et al. (2019) [39] | No work-related outcomes |
| Sandgren et al. (2000) [40] | No work-related outcomes |
| Schulman-Green et al. (2017) [41] | No work-related outcomes |
| Thompson et al. (2014) [42] | No work-related outcomes |
| Winick et al. (1977) [43] | Study design other than RCT or quasi-experimental design (with comparator). Non-controlled. |
