# Supplementary material for: Rehabilitation interventions to support return to work for women with breast cancer: a systematic review and meta-analysis
Source: BMC Cancer. 2021 Aug 5;21:895. doi: 10.1186/s12885-021-08613-x (PMC8340442; doi:10.1186/s12885-021-08613-x)
Supplement: Supplementary file 3 — Additional file 3. [file 12885_2021_8613_MOESM3_ESM.docx]

**SUPPLEMENTARY MATERIAL 3: Risk of bias assessment rationale**

| **Author** | **Bias** | | **Judgement** | **Support for Judgement** |
| --- | --- | --- | --- | --- |
| *Björneklett et al, 2013* | Selection Bias | Sequence generation | Low risk | Quote: “*Patients were stratiﬁed according to adjuvant chemotherapy and randomized in blocks of four by the use of closed envelopes”.* |
|  |  | Allocation concealment | Unclear risk | Comment: Sealed envelopes however does not state if sequentially numbered and/or opaque as per the SNOSE method (Sequentially Numbered, Opaque, Sealed Envelope). |
|  | Performance bias and detection bias | Blinding of participants and personnel | High risk | Comment: Impossible to blind participants due to the nature of the intervention. |
|  |  | Blinding of outcome assessors | Unclear risk | Comment: Participants were the outcome assessors (as using self-reported questionnaires). It is unclear if knowing which intervention they were randomised to, would have directly influenced the outcomes. |
|  | Attrition bias | Incomplete outcome data | High risk | Comment: Attrition rates outlined however no reasons for dropouts explained or mentioned in the analysis. |
|  | Reporting bias | Selective reporting | Low risk | Comment: Appear to present results for three main outcomes; Sick leave, Health care utilisation and Costs of healthcare. |
|  |  | Other biases | High risk | Comment: Potential weaknesses identified in the discussion; (i) Self-reported/Subjective outcome measures, which were (ii) not validated. Health status before randomisation unknown. |
| *Bolam et al, 2019* | Selection Bias | Sequence generation | Low risk | Comment: Computer-generated randomisation, as per original paper (Wengström et al, 2017) |
|  |  | Allocation concealment | Unclear risk | Comment: Unclear how concealment occurred. |
|  | Performance bias and detection bias | Blinding of participants and personnel | High risk | Comment: Impossible to blind participants due to the nature of the intervention. |
|  |  | Blinding of outcome assessors | Unclear risk | Comment: Participants were the outcome assessors (as using self-reported questionnaires). It is unclear if knowing which intervention they were randomised to, would have directly influenced the outcomes. |
|  | Attrition bias | Incomplete outcome data | High risk | Comment: There are multiple timepoints where there is lost to follow-up/dropouts, described in figure. There are many more dropouts in the usual care immediately post-randomisation than other group. Also, not all is clear e.g. from week 16 after usual care (n=182), number approached for 2-year follow-up down to n=179. No allowance for this is made in approach to analysis. |
|  | Reporting bias | Selective reporting | Low risk | Comment: Appears all pre-specified outcomes were reported |
|  |  | Other biases | Unclear risk | Comment: Potential sources of bias are that information on other activity being conducted by participants outside of the intervention was not complete a |
| **Author** | **Bias** | | **Judgement** | **Support for Judgement** |
| *Hubbard et al, 2013* | Selection Bias | Sequence generation | Low risk | Comment: Allocation sequence generated from a Bernoulli probability distribution with a specified probability of 0.5, which ensured participants had an equal chance of being in either group. |
|  |  | Allocation concealment | Low risk | Comment: Allocation sequence concealed from researcher. Administrator who was not involved in process, assigned participants to intervention and usual care groups. |
|  | Performance bias and detection bias | Blinding of participants and personnel | High risk | Comment: Impossible to blind participants due to the nature of the intervention. |
|  |  | Blinding of outcome assessors | Unclear risk | Comment: Participants were the outcome assessors (as using self-reported questionnaires). It is unclear if knowing which intervention they were randomised to, would have directly influenced the outcomes. |
|  | Attrition bias | Incomplete outcome data | Low risk | Comment: Intervention group was n=7 for final analysis (Down 1 participant from allocation). Control group was n=11 (down 3 participants from allocation). Reasons were provided for attrition. Have explained exclusions, however, could have used imputation methods in sensitivity analysis to determine the impact of missing data on results. |
|  | Reporting bias | Selective reporting | Low risk | Comment: Appears all pre-specified outcomes were reported |
|  |  | Other biases | High risk | Comment: While demographics and clinical characteristics are outlined for each group, it's not clear if there are any statistically significant differences between groups. Likely due to small sample. Other potential sources of bias identified in the discussion; Low sample size, potentially healthier sample and those with greatest need may be excluded. |
| *Ibrahim et al, 2017* | Selection Bias | Sequence generation | Low risk | Comment: Block randomisation used. |
|  |  | Allocation concealment | Unclear risk | Comment: Unclear who individual was in the allocation process. The treatment assignment was hidden until name entered but not explained how hidden? |
|  | Performance bias and detection bias | Blinding of participants and personnel | High risk | Comment: Impossible to blind participants due to the nature of the intervention. |
|  |  | Blinding of outcome assessors | Unclear risk | Comment: Participants were the outcome assessors (using self-reported questionnaires). It is unclear if knowing the intervention they were randomised to, would have directly influenced outcomes. |
|  | Attrition bias | Incomplete outcome data | Unclear risk | Comment: Two dropped out and 3 died during the study. No explanation how/if they were included in the analysis. Table 1 showing baseline and follow-up does not provide numbers in final analysis. |
|  | Reporting bias | Selective reporting | Low risk | Comment: Appears all pre-specified outcomes were reported |
|  |  | Other biases | High risk | Comment: Higher baseline levels of activity in exercise vs control group. Limited time available for intervention, potential for selection bias. Lack of adherence with self-reported logs |
| **Author** | **Bias** | | **Judgement** | **Support for Judgement** |
| *Jong et al, 2018* | Selection Bias | Sequence generation | Low risk | Comment: Described adequately using blocked randomisation |
|  |  | Allocation concealment | Unclear risk | Comment: While it is reported that the study monitor was blinded for allocation sequence, it is unclear how this achieved i.e. through sequential sealed envelopes, etc. |
|  | Performance bias and detection bias | Blinding of participants and personnel | High risk | Comment: Impossible to blind participants due to the nature of the intervention. |
|  |  | Blinding of outcome assessors | Unclear risk | Comment: Participants were the outcome assessors (as using self-reported questionnaires). It is unclear if knowing which intervention they were randomised to, would have directly influenced the outcomes. |
|  | Attrition bias | Incomplete outcome data | High risk | Comment: Not all participants who were randomised were accounted for at 3 months since the reasons provided do not add to the total dropping out. |
|  | Reporting bias | Selective reporting | Low risk | Comment: Appears all pre-specified outcomes were reported |
|  |  | Other biases | High risk | Comment: Intervention group had significantly more participants who had not received hormone therapy. Many outcomes were psychological which may have been impacted by the fact that there was a statistically significant difference between groups by hormone therapy. |
| *Maguire et al, 1983* | Selection Bias | Sequence generation | Low risk | Comment: Sequence generated using a random numbers table. |
|  |  | Allocation concealment | Unclear risk | Comment: Unclear who completed the randomisation. |
|  | Performance bias and detection bias | Blinding of participants and personnel | High risk | Comment: Impossible to blind participants due to the nature of the intervention. |
|  |  | Blinding of outcome assessors | Unclear risk | Comment: Participants were the outcome assessors (as using self-reported questionnaires). It is unclear if knowing which intervention they were randomised to, would have directly influenced the outcomes. |
|  | Attrition bias | Incomplete outcome data | Low risk | Comment: Reported numbers at each stage, including numbers of drop-outs and reasons for drop out. There were a small number (20/172) of those who were excluded from the analysis for different reasons leaving 152. All 152 were accounted for in the tables presented |
|  | Reporting bias | Selective reporting | Low risk | Comment: Based on the outcomes they appear to describe all outcomes are presented, no evidence of selective reporting |
|  |  | Other biases | Unclear risk | Comment: Participant characteristic - unclear regarding baseline characteristics and if there were any statistically significant differences between groups. Paper reports that the group 'proved closely matched on variables', but no data reported to support this. As the authors do not present any limitations to the study it is not clear if other sources of bias might exist |
| **Author** | **Bias** | | **Judgement** | **Support for Judgement** |
| *Maunsell et al, 1996* | Selection Bias | Sequence generation | Low risk | Comment: Use of sealed envelopes (prepared using a random numbers table) randomly varied block sizes used. |
|  |  | Allocation concealment | Low risk | Comment: Nurse was blinded to patient's treatment allocation, where the secretary randomised all patients. |
|  | Performance bias and detection bias | Blinding of participants and personnel | High risk | Comment: Impossible to blind participants due to the nature of the intervention. |
|  |  | Blinding of outcome assessors | Unclear risk | Comment: Participants were the outcome assessors (as using self-reported questionnaires). It is unclear if knowing which intervention they were randomised to, would have directly influenced the outcomes. |
|  | Attrition bias | Incomplete outcome data | Low risk | Comment: Attrition rates outlined and explanation provided. |
|  | Reporting bias | Selective reporting | Low risk | Comment: Appears all pre-specified outcomes were reported |
|  |  | Other biases | Unclear risk | Comment: Participant characteristics - No p values available to confirm there were no statistically significant differences between groups. Only one site used so potential bias in that regard. |
| *Mourgues et al, 2014* | Selection Bias | Sequence generation | Unclear risk | Comment: While it is reported that randomisation was balanced and stratified, it is unclear as to how this was done. |
|  |  | Allocation concealment | Unclear risk | Comment: Unclear if allocation was concealed from team members. Randomisation performed by the oncology centre is vague. |
|  | Performance bias and detection bias | Blinding of participants and personnel | High risk | Comment: Impossible to blind participants due to the nature of the intervention. |
|  |  | Blinding of outcome assessors | Unclear risk | Comment: Participants were the outcome assessors (as using self-reported questionnaires). It is unclear if knowing which intervention they were randomised to, would have directly influenced the outcomes. |
|  | Attrition bias | Incomplete outcome data | Low risk | Comment: While there are no reasons provided for attrition levels of the three analysis timepoints, the authors do account for missing data in the analysis using a mixed model (MRMs). |
|  | Reporting bias | Selective reporting | Low risk | Comment: Appears all pre-specified outcomes were reported |
|  |  | Other biases | Unclear risk | Comment: Participant characteristics - Statistically significant difference between the two groups (p <0.05) for monthly activities where control group complete 132.8 hours per month vs. intervention group with 99.5 hours. All other variables tested had no statistically significant difference. Unclear if this had influence on outcomes. |
| **Author** | **Bias** | | **Judgement** | **Support for Judgement** |
| *Rogers et al, 2009* | Selection Bias | Sequence generation | Low risk | Quote: “*Participants were randomized after completion of all base- line assessments. Randomization was computer generated and kept in sealed envelopes until randomization to prevent bias in group allocation by study personnel.”* |
|  |  | Allocation concealment | Unclear risk | Comment: Randomised allocation kept in sealed envelopes to prevent bias in group allocation by study personnel. However, no mention if envelopes were opaque or sequentially numbered. As per SNOSE technique, envelopes should be Sequentially Numbered, Opaque, Sealed Envelope. |
|  | Performance bias and detection bias | Blinding of participants and personnel | High risk | Comment: Impossible to blind participants due to the nature of the intervention. |
|  |  | Blinding of outcome assessors | Unclear risk | Comment: Participants were the outcome assessors (as using self-reported questionnaires). It is unclear if knowing which intervention they were randomised to, would have directly influenced the outcomes. |
|  | Attrition bias | Incomplete outcome data | Low risk | Comment: The authors provide an assessment of missing data in the statistical analysis section (for individual items on scales and imputation) and for those lost to follow-up. However, the numbers on each outcome are not provided in the tables so assumed all others were complete or imputed. |
|  | Reporting bias | Selective reporting | Low risk | Comment: Appears all pre-specified outcomes were reported |
|  |  | Other biases | High risk | Comment: Potentially other sources of bias, including variation in stage/time since diagnosis, short follow-up time. As this is a feasibility study, it is not powered for effects. |
